# Case report: Regression after low-dose glucocorticoid therapy in a case of acute immune myocarditis induced by anti-PD-1 therapy for NSCLC

**DOI:** 10.3389/fonc.2024.1404045

**Published:** 2024-05-24

**Authors:** Liqianqi Chen, Suihao Zhang, Long Gong, Yucong Zhang

**Affiliations:** ^1^ The Second Clinical Medical College, Jinan University, Shenzhen, China; ^2^ Department of Cardiology, Shenzhen People’s Hospital, Jinan University, Shenzhen, Guangdong, China; ^3^ Department of Radiotherapy, Shenzhen People’s Hospital, Jinan University, Shenzhen, Guangdong, China

**Keywords:** PD-1 inhibitor, immune myocarditis, non-small cell lung cancer, low-dose glucocorticoid, methylprednisolone

## Abstract

**Background:**

PD-1 inhibitors exhibit efficacy in managing unresectable/metastatic driver gene-negative NSCLC, albeit with potential immune-related adverse events (irAEs). Among these, immune checkpoint inhibitor-associated myocarditis (ICI-M) is rare yet lethal. This study presents the initial successful instance of ICI-M in a lung cancer patient, rescued by low-dose glucocorticoids post-deterioration during treatment.

**Case summary:**

A 78-year-old male with a medical history of stage IV pT3N2M1 NSCLC underwent four cycles of palliative chemotherapy, resulting in stable disease (SD). Subsequent to declining further chemotherapy, the patient was transitioned to a targeted therapy regimen comprising Anlotinib in conjunction with PD-1 inhibitor immunotherapy. On the 26th day post-administration of the PD-1 inhibitor, the patient manifested Grade 2 immune-mediated myocarditis. Treatment encompassing 1mg/kg methylprednisolone combined with immunoglobulin shock therapy was initiated for 3 days, achieving symptomatic control. Nonetheless, upon tapering methylprednisolone dosage to 4–8mg/3–4d, the condition deteriorated, necessitating transfer to the intensive care unit. Methylprednisolone dosage was escalated to 80mg/day for 3 days, followed by gradual reduction by one-third to two-thirds weekly, culminating in the patient’s safe discharge from the hospital.

**Conclusion:**

Immune-related myocarditis linked to checkpoint inhibitors is often managed effectively with high-dose glucocorticoid therapy. However, in Asian populations, low-dose glucocorticoids are increasingly utilized for salvage therapy, yielding favorable outcomes and improving prognosis compared to European populations.

## 1 Introduction

Immune checkpoint inhibitors (ICIs) represent a significant advancement in cancer therapy in recent years. Primarily, their mechanism entails the inhibition of immune checkpoint activity via antibodies, thereby restoring and augmenting effector T lymphocyte function to selectively identify and eradicate tumor cells (1). PD-1 is a prototypical agent among ICIs. Nonetheless, treatment with PD-1 monoclonal antibodies targeting tumor cells may paradoxically exacerbate autoimmune responses, leading to immune-related adverse events including hypothyroidism, rash, myocarditis, pneumonitis, and enteritis.

ICI-M is classified as a rare immune-related adverse reaction, distinguished by early onset, nonspecific symptoms, rapid progression, and high mortality rates (2–5). The prevalence of ICI-M varies from 0.06% to 3.8%, with severe myocarditis occurring in approximately 0.09% of cases (3, 5, 6). Its clinical manifestations are varied, presenting typical symptoms including malaise, chest pain, anxiety, and dyspnea, which may progress to respiratory distress, and in severe cases, cardiogenic shock and sudden death.

This article reports a case of an elderly patient with advanced lung adenocarcinoma who developed ICI-M after treatment with the PD-1 inhibitor sintilimab. In terms of hormone dosage, there is a controversy between Chinese and European guidelines. 2021 CSCO related guidelines recommend an initial methylprednisolone dose of 1–4 mg/kg/d, and ESMO of the European Society of Medical Oncology believes that when myocarditis is suspected, high-dose shock therapy with methylprednisolone 0.5–1 g/d for 3–5 days should be given, which may be related to hormone sensitivity. This case demonstrates that early low-dose hormones are also effective benefits in salvage therapy for immune-related myocarditis in Asian populations compared to high-dose shock therapy in European Europe.

## 2 Case description

**Figure 1:**
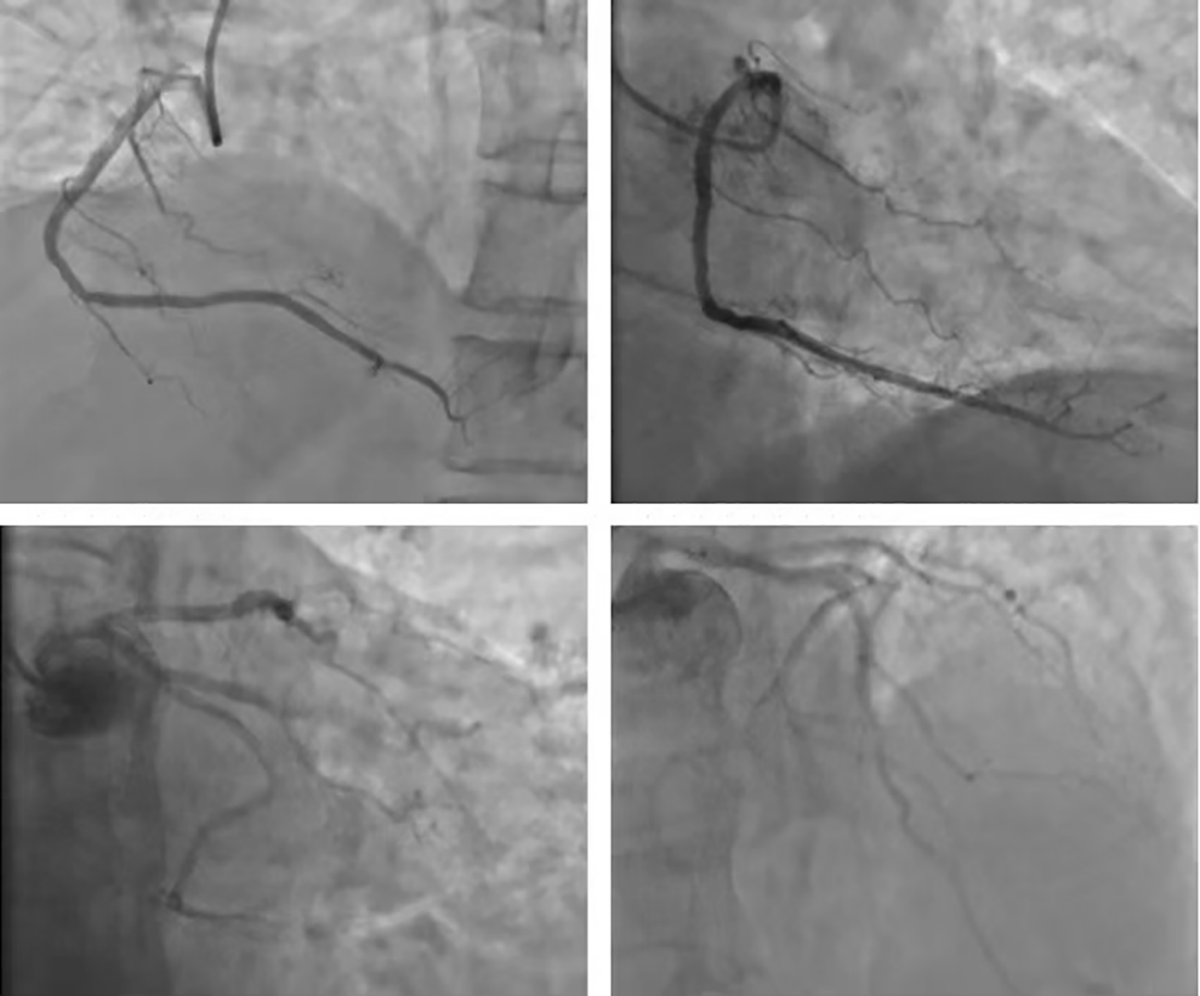
Coronary arterial photovation: coronary blood supply right advantage type; LM normal, LAD arteriosclerosis; remote blood flow TIMI 3; LCX arteriosclerosis, remote blood flow TIMI level 3; RCA normal arteriosclerosis, remote blood flow TIMI level 3 level 3 level 3, ACS evidence is not sufficient.

The patient, a 78-year-old male, underwent “Thoracoscopic radical treatment of right upper lung cancer with pleural adhesion branding and intercostal nerve closure” in March 2021. Postoperative pathology revealed poorly differentiated adenocarcinoma. Follow-up chest CT evaluation indicated pathological complete response (pCR). MRI of both hips suggested the possibility of left iliac metastasis. The AJCC staging was determined as pT3N2Mx, corresponding to stage IIIB. The patient received four cycles of postoperative palliative care, with evaluation indicating stable disease (SD). Subsequently, the patient declined further chemotherapy and was prescribed Anlotinib targeted therapy. In December 2021, PET-CT scan revealed left iliac metastasis, and the diagnosis was updated to pT3N2M1, corresponding to stage IVB. On December 20, 2021, the patient received the first cycle of sintilimab treatment at a dose of 200 mg/dose q3w. The baseline assessment of cardiac enzymes, thyroid function, cortisol, adrenocorticotropic hormone, and electrocardiogram were unremarkable. The patient received the second cycle of sintilimab at the same dosage on January 10, 2022. However, on January 16, 2022, the patient was admitted to the hospital with complaints of “chest tightness and dyspnea”. The patient had a history of hypertension for more than 1 year, with a peak blood pressure of 150 mmHg/110 mmHg, which remained untreated. The patient denied any history of diabetes mellitus or coronary artery disease, and had no recent history of upper respiratory tract infections or exposure to toxins or radioactive substances. Chest CT revealed postoperative alterations in the right lung consistent with previous findings. The electrocardiogram demonstrated corresponding ST segment changes in select leads. Troponin I levels were elevated at three times the normal range, myoglobin levels were elevated at 29 times the normal range, creatine kinase levels were elevated at 19 times the normal range, creatine kinase isoenzyme levels were elevated at 8 times the normal range, lactate dehydrogenase levels were elevated at 3 times the normal range, while amino-terminal brain natriuretic peptide precursors pro-BNP and D-dimer levels were within normal limits. Echocardiogram combined with myocardial perfusion imaging revealed normal ejection fraction, with no significant abnormalities in left ventricular wall motion or ventricular diastolic function, and normal left ventricular myocardial perfusion. Coronary angiography demonstrated a right dominant coronary artery distribution pattern with a normal left main coronary artery and atherosclerosis in the left anterior descending artery (LAD) with TIMI grade 3 distal flow. Atherosclerosis was also noted in the left circumflex artery (LCX) with TIMI grade 3 distal flow, while the right coronary artery (RCA) exhibited normal anatomy with TIMI grade 3 distal flow. There was insufficient evidence of acute coronary syndrome (ACS) based on these findings (**Figure 1**).

In conjunction with the pertinent examinations, immune-related myocarditis G2 with class II cardiac function was considered. The patient’s weight was 50 kg. Immediate administration of methylprednisolone 40 mg for 4 days in combination with immunoglobulin 20 mg for 4 days in accordance with the relevant 2021 CSCO guidelines (initial methylprednisolone dose 1–4 mg/kg/d). The patient’s symptoms improved significantly, and creatine kinase decreased by 84%, creatine kinase isoenzyme decreased by 70%, lactate dehydrogenase decreased by 33%, and myoglobin decreased by 42%. The dosage was reduced to methylprednisolone 32mg/d for 4 days, and then reduced to 28mg/d after the symptoms stabilized. Subsequently, the patient manifested recurrent chest tightness and dyspnea, indicative of exacerbated symptoms, and was subsequently diagnosed with Type 2 respiratory failure accompanied by respiratory acidosis. Troponin levels exhibited an elevation compared to previous measurements, necessitating transfer to the intensive care unit for initiation of mechanical ventilation to address the respiratory insufficiency. Concurrently, hormonal therapy was intensified to a dosage of 40mg/d. However, clinical manifestations persisted unabated, with negligible alterations observed in certain cardiac enzymes, and troponin levels remained elevated. Transthoracic echocardiography revealed no notable abnormalities in ventricular wall motion at rest and preserved left ventricular systolic function.

**Figure 2:**
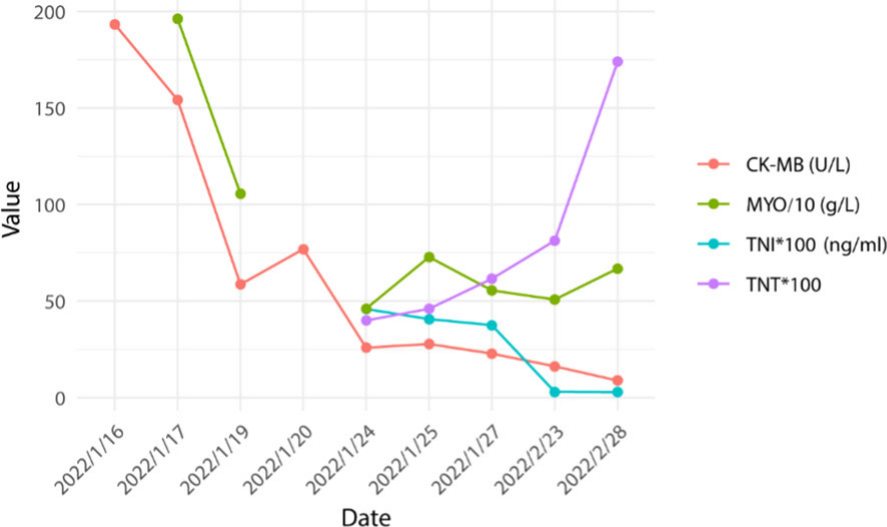
Trend of myocardial enzyme changes.

**Figure 3:**
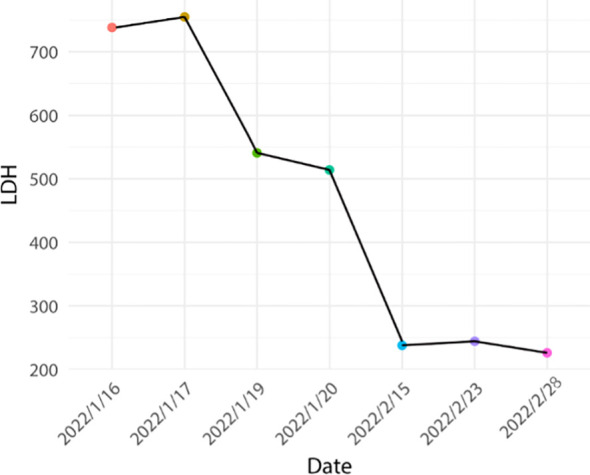
Trend of lactate dehydrogenase changes.

**Figure 4:**
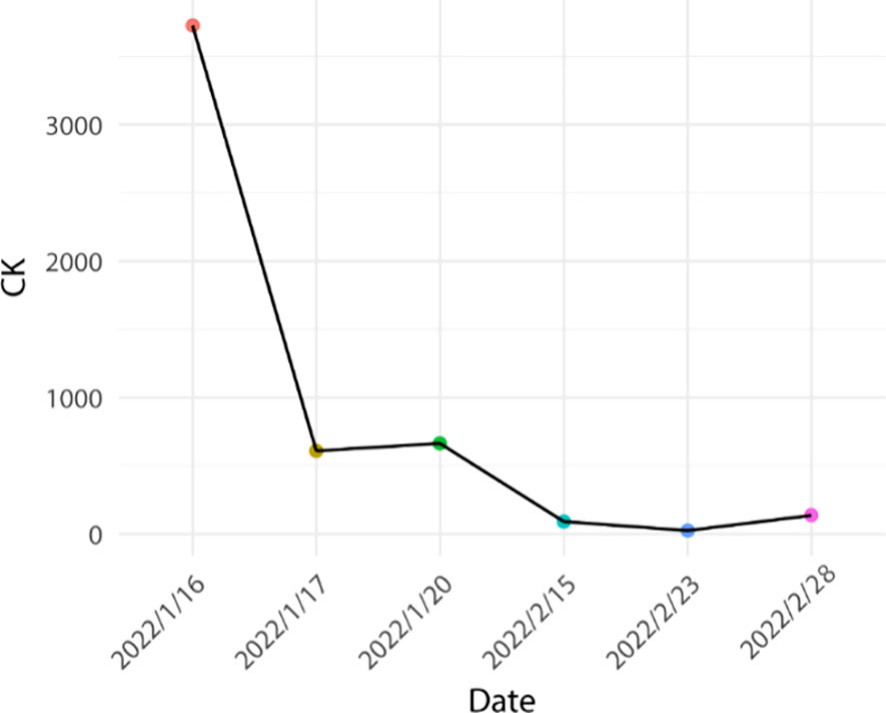
Trend of creatine kinase changes.

**Figure 5:**
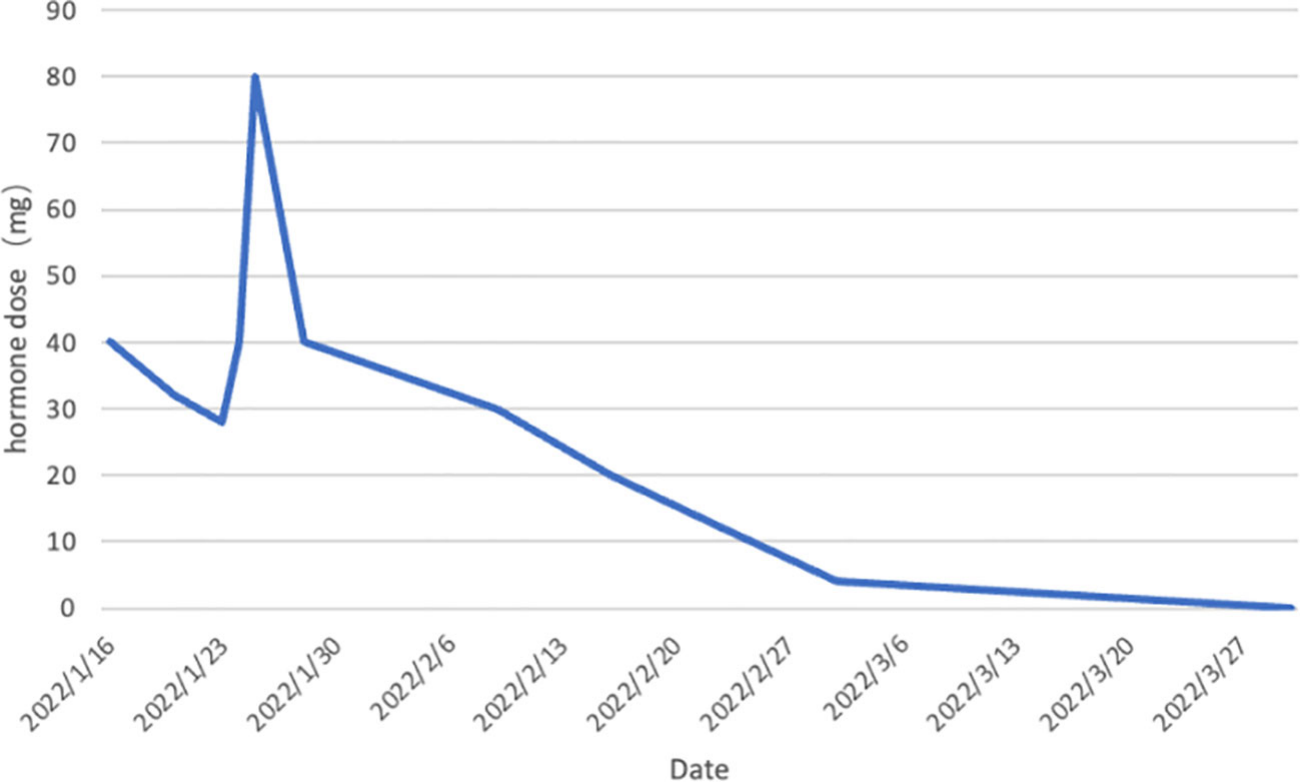
Trend of hormone dose changes. Prednisone was first administered on January 16, 2022 at 40mg.The dose was reduced to 32mg on January 20.The dose was reduced to 28mg on January 23.The patient’s condition deteriorated and she was transferred to the ICU on January 24.The dose was adjusted upward to 40mg.The dose was re-adjusted to 80mg on January 25.The dose was reduced in steps until the patient’s condition stabilized and she was transferred to the ICU on February 9.The dose was reduced to 5mg on March 2.The patient was discharged on March 30.The drug was discontinued on March 30.The patient’s condition stabilized and he was discharged from the hospital. stabilized and was discharged from the hospital. The drug was discontinued on March 30th. (5mg prednisone is equivalent to 4mg methylprednisolone).

Based on the aforementioned information, progression of ICI-M from Grade 2 to Grade 3 was acknowledged, leading to adjustments in the treatment protocol. Methylprednisolone at a dose of 80mg/d was administered for a duration of 3 days alongside immunoglobulin at a dosage of 20mg/d, also for 3 days. Subsequently, the dosage was tapered to 40mg/d of methylprednisolone for 12 days, with subsequent reductions of 10mg/d every week. After two weeks, the dose was changed to 15 mg/d prednisone tablets for one week. Final monitoring demonstrated a 95% decrease in troponin I levels, a 66% decrease in myoglobin levels, a 98% decrease in creatine kinase levels, a 95% decrease in creatine kinase isoenzyme levels, and a 20% decrease in lactate dehydrogenase (**Figures 2**–**4**). Then the patient was safely stabilized and discharged to continue 5 mg/d prednisone tablets for a month (**Figure 5**). (5mg prednisone is equivalent to 4mg methylprednisolone).

Continued follow-up has been conducted via telephone, with the patient reporting no recurrence of symptoms such as chest tightness, dyspnea, or shortness of breath. Overall, the patient is capable of performing daily activities with minimal assistance.

## 3 Discussion

In 2020, there are projected to be an estimated 2.2 million incident cases of lung cancer and 1.8 million deaths attributable to this disease. Non-small cell lung cancer (NSCLC) represents over 85% of all lung cancer cases, with adenocarcinoma being the most prevalent histologic subtype, accounting for 40% of cases (7). In recent years, the advancement of immunotherapy in the field of lung cancer research has resulted in significant prolongation of patient survival. However, the associated adverse effects should not be underestimated. Among immune-related adverse events (irAEs), immune myocarditis is the rarest yet most deadly, with myocarditis carrying a mortality rate of 40% to 50% (5, 8, 9). A case report published in the New England Journal of Medicine in 2016 unveiled, for the first time, two instances of fulminant and fatal myocarditis induced by PD-1 monoclonal antibody (2). This discovery garnered global attention. The frequency of immune checkpoint inhibitors (ICIs)-associated myocarditis varies from 0.06% to 3.8%, with severe myocarditis occurring in approximately 0.09% of cases, and a median onset time of 17–34 days. Some patients experience severe myocarditis after receiving only 1–2 doses of ICIs, predominantly within 3 months of initiation of therapy (3, 5, 6). The elevated rates of morbidity and mortality associated with myocarditis present a significant challenge to both physicians and patients.

PD-1, an immunomodulatory checkpoint receptor expressed on activated T cells’ surface, engages with its ligand, inducing T cell dysfunction. This mechanism serves as a crucial regulator of immune tolerance in peripheral tissues and areas of chronic inflammation (10, 11). Inhibiting PD-1 or its ligands with PD-1/PD-L1 inhibitors activates dormant T cells, enhancing tumor cell clearance while potentially triggering non-specific targeting of healthy cells by activated T cells, thereby predisposing to immune-related adverse events (12). The occurrence of prevalent T-cell receptor sequences with high frequency in cardiac, skeletal muscle, and tumor tissues of patients experiencing ICI-M (2, 13–15), along with the observed imbalance in PD-1 to PD-L1 expression induced by immune checkpoint inhibitors, thereby impeding the binding of PD-1/PD-L1 to the discordant ligand, may constitute precipitating elements in myocardial autoimmune reactions (16).

The varied clinical presentations of PD-1-induced ICI-M, often featuring asthenia, angina, anxiety, and dyspnea, alongside its nonspecific nature and the absence of systolic impairment in half of the cases (3), render it prone to underrecognition, contributing to a considerably elevated rate of misdiagnosis of PD-1-induced ICI-M in the initial phases of immunotherapeutic intervention. Furthermore, alternative forms of cardiomyopathy that bear resemblance to myocarditis have been linked to ICIs therapy, including Takotsubo syndrome (17).

There are currently no established standardized diagnostic criteria for PD-1-induced ICI-M. In this scenario, coronary angiography was employed to exclude acute coronary syndrome (ACS). Subsequently, endomyocardial biopsy is considered the most precise diagnostic modality for immune myocarditis (18), but its utility is limited due to invasiveness and potential complications. In order to optimize the management of patients with ICI-M, the CSCO Guidelines for the Management of Toxicity Associated with Immune Checkpoint Inhibitors classify myocarditis into 4 categories based on the following criteria: Grade 1 (G1): Elevated biomarkers of cardiac injury without accompanying cardiovascular symptoms, ECG alterations or echocardiographic abnormalities. Grade 2 (G2): Mild cardiovascular symptoms with concurrent abnormalities in cardiac injury biomarkers and ECG, but no severely deranged echocardiographic findings. Grade 3 (G3): Markedly abnormal cardiac biomarkers and severely disturbed ECG/UCG, accompanied by significant symptoms at rest or upon minimal exertion. Grade 4 (G4): Severe symptoms with hemodynamic instability, life-threatening presentation requiring urgent medical intervention.

Numerous studies have demonstrated that glucocorticoids represent the optimal therapeutic approach for immune checkpoint inhibitor-related cardiotoxicity (5, 19–21). Importantly, existing evidence indicates that the administration of hormonal treatment for immune-related adverse events associated with ICIs does not compromise the efficacy of ICIs. Alternative therapies such as anti-CD3 antibodies, CTLA-4 agonists, and anti-CD52 antibodies exist; however, there is a lack of pertinent clinical trials validating their efficacy (21). Prophylactic glucocorticoid administration in PD-1/PD-L1 patients may attenuate the antitumor effectiveness of immune checkpoint inhibitor drugs, thus prophylactic glucocorticoid use is discouraged. Furthermore, in cases of hormonal resistance development, intensive immunosuppression or second-line immunosuppressive protocols should be deliberated (21).

In this case, we postulate that the exacerbation of the patient’s condition is correlated with inadequate maintenance duration during dose reduction, failing to impede further disease progression. As a recommendation, patients with ICI-M should be managed at the upper threshold of the dosing interval for maintenance duration (22, 23). The European Society of Medical Oncology (ESMO) suggests that when myocarditis is suspected, administration of a higher dose of methylprednisolone at 0.5–1 g/d for a period of 3–5 days can be considered (21). Variability in glucocorticoid sensitivity among distinct populations may exist(24), as demonstrated in this case where the patient achieved therapeutic efficacy comparable to that in the European population while following the dosage outlined in the CSCO guideline (significantly lower than the ESMO guideline). A systematic analysis of case reports of immune checkpoint inhibitor-associated myocarditis (24) showed that the most commonly used hormone dose for the treatment of immune myocarditis in China is 1–2 mg/kg/d, i.e., the low-dose hormones described in this article, and that similar use was made in two case reports of immune checkpoint inhibitor-associated hepatitis (25) and immune checkpoint inhibitor-associated rheumatic polymyalgia (26), both of which showed promising results. Concurrently, the patient was able to mitigate hormone-related adverse events such as femoral head necrosis, gastrointestinal bleeding, and secondary infections to some degree.

In individuals with previous immune-mediated myocarditis, there appears to be a modest advantage in reinitiating the same immunotherapy once markers indicating myocardial injury have normalized (27). However, the safety of this approach has not been definitively established, and there is presently a scarcity of data regarding this matter.

## 4 Conclusion

The onset of PD-1-induced ICI-M is exceedingly prompt, and once it is suspected that a patient is experiencing ICI-M, immunotherapies should be halted without delay, and a regimen of high-dose glucocorticoids should be initiated in accordance with the severity of symptoms and testing markers. Importantly, this instance illustrates that early implementation of low-dose glucocorticoid rescue therapy in the initial stages of immune myocarditis and gradual tapering can still achieve therapeutic efficacy that is not inferior to that of high-dose glucocorticoid rescue therapy. The utilization of low-dose glucocorticoids for the rescue of immune myocarditis represents a complex and relatively uncharted domain, potentially associated with variations in hormone regulation mechanisms among diverse populations. Further investigation, facilitated by ongoing technological advancements and research endeavors, is imperative to enhance our comprehension of the disease and optimize therapeutic strategies.

## Data availability statement

The original contributions presented in the study are included in the article/supplementary material. Further inquiries can be directed to the corresponding authors.

## Ethics statement

The studies involving humans were approved by Ethics Committee of Shenzhen People’s Hospital. The studies were conducted in accordance with the local legislation and institutional requirements. Written informed consent for participation was not required from the participants or the participants’ legal guardians/next of kin in accordance with the national legislation and institutional requirements. Written informed consent was obtained from the individual(s) for the publication of any potentially identifiable images or data included in this article.

## Author contributions

LC: Writing – original draft. SZ: Writing – original draft. LG: Writing – review & editing. YZ: Writing – review & editing.
